# Effect of a Multimedia Patient Decision Aid to Supplement the Informed Consent Process of a Peripherally Inserted Central Venous Catheter Procedure: Pre-Post Quasi-Experimental Study

**DOI:** 10.2196/11056

**Published:** 2018-11-28

**Authors:** Azizeh K Sowan, Arlienita R Beraya, Adrian Carrola, Charles Reed

**Affiliations:** 1 School of Nursing University of Texas Health at San Antonio San Antonio, TX United States; 2 University Health System San Antonio, TX United States

**Keywords:** multimedia, central venous catheters, decision support techniques, informed consent, intensive care units

## Abstract

**Background:**

Informed consent is a complex process to help patients engage in care processes and reach the best treatment decisions. There are many limitations to the conventional consent process that is based on oral discussion of information related to treatment procedures by the health care provider. A conclusive body of research supports the effectiveness of multimedia patient decision aids (PtDAs) in the consent process in terms of patient satisfaction, increased knowledge about the procedure, reduced anxiety level, and higher engagement in the decision making. Little information is available about the effectiveness of multimedia PtDAs in the consent process of invasive therapeutic procedures such as the peripherally inserted central venous catheter (PICC).

**Objective:**

The objective of this study was to examine the effectiveness of a multimedia PtDA in supplementing the consent process of the PICC for patients in 10 acute and intensive care units in terms of knowledge recall, knowledge retention, satisfaction with the consent process, and satisfaction with the PICC multimedia PtDA.

**Methods:**

This pre-post quasi-experimental study included 130 patients for whom a PICC was ordered. Patients in the control group (n=65) received the conventional consent process for the PICC, while those in the intervention group (n=65) received the multimedia PtDA to support the consent process of a PICC. All patients were surveyed for knowledge recall and retention about the procedure and satisfaction with the consent process. Patients in the intervention group were also surveyed for their satisfaction with the multimedia PtDA.

**Results:**

Compared with the control group, patients in the intervention group scored around 2 points higher on knowledge recall (*t_125_*=4.9, *P*<.001) and knowledge retention (*t_126_*=4.8, *P*<.001). All patients in the intervention group were highly satisfied with the multimedia PtDA, with a mean score of >4.5 out of 5 on all items. Items with the highest mean scores were related to the effect of the multimedia PtDA on knowledge retention (mean 4.9 [SD 0.2]), patient readiness to learn (mean 4.8 [SD 0.5]), complete understanding of the procedure-related complications (mean 4.8 [SD 0.4]), and patient role in maintaining the safety of the PICC (mean 4.8 [SD 0.5]). Patients in the two groups were highly satisfied with the consent process. However, 15% (10/65) patients in the control group reported that the following information was omitted from the discussion: patient and provider roles in the safety of the PICC, other treatment options, and common side effects. Furthermore, 2 patients commented that they were not ready to engage in the discussion.

**Conclusions:**

The multimedia PtDA is an effective standardized, structured, self-paced learning tool to supplement the consent process of the PICC and improve patient satisfaction with the process, knowledge recall, and knowledge retention.

## Introduction

Informed consent is a complex process that aims to discuss with patients complete, clear, and easy-to-understand information about a medical procedure, its treatment indications, associated risks and benefits, and other treatment alternatives using a two-way communication with a teach-back mechanism. Signing the consent form by the patient does not always indicate the process was informed and does not cover the health care team members from liability or protect them against lawsuits. There are numerous challenges related to informed consent, and they may be patient related (ie, special situations such as pain and discomfort, health literacy issues), clinician related (ie, concerns of providing extra information), workflow related (ie, time pressure and workload), organizational culture related (ie, lack of understanding the implications of the informed consent, lack of clear policies and procedures), or resources related (ie, lack of appropriate patient educational tools and decision aids to supplement the consent process).

The consent process is traditionally based on oral discussion of information related to treatment procedures by the health care provider. The effectiveness of this method is questionable given its unstructured and unstandardized nature and the differences in patients’ information needs [1,2]. To improve the conventional consent process, multimedia patient decision aids (PtDAs) have been heavily utilized as supplemental educational tools. A conclusive body of research supports the effectiveness of multimedia PtDAs in the consent process for patients undergoing surgeries in terms of higher patient satisfaction with the consent process, increased knowledge about surgeries, reduced anxiety level, and higher patient engagement in the decision-making process [3-10]. On the other hand, little information is available about the effectiveness of multimedia PtDAs in the consent process of invasive therapeutic procedures such as the peripherally inserted central venous catheter (PICC) [11]. Unlike elective surgeries and other medical procedures, when a PICC is ordered, it is often required for patients’ condition with little to no other treatment options. The consent process in this case should focus on discussing the procedure with the patient regarding its indications and associated risks, understanding the patient’s cultural values that may affect patient acceptance of the procedure, and highlighting the patient role in the safety of the PICC. In this study, we aimed to examine the effectiveness of a multimedia PtDA to supplement the consent process of the PICC for patients in acute care units (ACUs) and intensive care units (ICUs).

PICC is a very common invasive procedure for prolonged administration of medications, parenteral fluids, and blood products. On the other hand, this procedure is a major source for central line-associated bloodstream infections (CLABSIs) [12], central catheter-associated thrombosis [13-15], and other major life-threatening complications, including death. Therefore, patients should play an integral role in maintaining the safety of a PICC and minimizing its complications when competent.

In a previous study, we described the process of developing and “alpha” testing a multimedia PtDA for the PICC following a multidisciplinary, patient-centered, and systematic process [16]. The process was based on the Agency for Healthcare Research and Quality’s (AHRQ) Guide for Making Informed Consent an Informed Choice [17], the AHRQ’s Health Literacy Universal Precautions Toolkit Guide [18], and the AHRQ’s Patient Education Materials Assessment Tool Guide for Audio/Video Materials [19]. The PtDA was evaluated using the PtDAs’ quality criteria developed by the International Collaboration for PtDA Standards [20]. The PtDA multimedia program was delivered via the Interactive Patient Care solution GetWell Inpatient, which allows competent patients and family members to review the program as many times as they want from the patient room and when they are ready to do so. The PtDA included information about the purpose of engaging patients and family members in care processes, a disclaimer, and PICC-related content that includes a definition of the PICC, indications, possible insertion sites, other treatment options, risks and complications associated with a PICC, steps of the procedure (before, during, and after a PICC), patient and health care team roles in the care and safety of a PICC during hospitalization, expected time period for having the catheter, safety issues when a patient leaves the hospital with a PICC, and a conclusion. This study describes a “beta” testing of the PICC multimedia PtDA to enhance our understanding of the effectiveness of this tool in practice.

## Methods

### Design, Setting, and Sample

This pre-post quasi-experimental study was conducted in a university teaching hospital in the Southwest of the United States after obtaining the institutional review board approval. The study included 130 patients from all inpatient units where a PICC was ordered. These include 5 ACUs (2 medical-surgical and 3 surgical), 1 hematology and oncology unit, and 4 ICUs (3 surgical and 1 medical). The preintervention phase included a convenient sample of 65 patients who received the conventional consent process for the PICC, and the post phase included 65 patients who received the multimedia PtDA to support the consent process of a PICC. Only competent patients (ie, those with absence of dementia) were included. Patients were excluded if they had a PICC before this hospitalization, were health care professionals, or had a current diagnosis of depression or anxiety disorders. The sample size was determined on the basis of a desirable improvement in knowledge retention (one of the study outcomes as described below) about the PICC by at least 50%, equal number of patients in the 2 groups, an alpha level of .05, and a beta level of .80. Given these conditions, the enrollment of at least 65 patients in each group should be achieved.

### Description of the Consent Process

The PICC consent process starts with the provider who discusses the need for a PICC with the patient and places an order in the electronic medical record (EMR). In the conventional process, a nurse from the vascular access team visits the patient to discuss the procedure details and obtain the consent form. After the discussion and answering the patient’s questions, the patient signs a Web-based iMedConsent form for a PICC using an iPad. A certified interpreter is consulted for non-English-speaking patients.

After the creation of the multimedia PtDA for the PICC, the PtDA was integrated into the EMR and delivered to patients using GetWell Inpatient [16]. GetWell Inpatient is a patient education and entertainment system that is integrated into the EMR and used to distribute and track the use of educational videos and multimedia programs in addition to other purposes related to engaging patients in care processes [16]. The use of the PICC multimedia PtDA to supplement the PICC consent process resulted in workflow redesign, as described previously [16]. One of the major changes was related to the new coordination between bedside nurses from the ACUs and ICUs and nurses from the vascular access team. Bedside nurses from the ACUs and ICUs were tasked to make sure that the patient watched the PICC multimedia PtDA within a reasonable timeframe after placing the PICC order in the EMR and before the vascular access team visits the patient for the teach-back and answering questions. The multimedia PtDA was developed in English and Spanish languages [16]. The version watched by the patient was based on his or her preferred language.

As part of standardizing the process during the alpha testing of the product [16], we trained all 6 nurses from the vascular access team and all bedside nurses from the ACUs and ICUs on the new workflow and observed 12 consent processes (2 per nurse from the vascular access team) to ensure adherence to the new workflow. In addition, 2 ICU nurse educators observed the 12 consent processes to ensure that nurses follow a standardized consent process. As mentioned before, helping a patient watch the program was assigned to bedside nurses. Based on the observations’ results, all eligible patients watched the PtDA to learn about the procedure before signing the consent form, and family members also watched the program when they were available with patients. The only issue revealed by the observations was lack of clarity about who was supposed to ensure that the patient watched the video (bedside nurses versus vascular access team). Thus, further training was provided, and roles and responsibilities in the new workflow were emphasized.

### Measurement and Instrumentation

The main study outcomes were knowledge recall, knowledge retention, and patient satisfaction with the consent process. These are the same outcomes examined in the alpha testing process of the PICC multimedia PtDA [16]. The alpha testing study provided a detailed description of the instruments used to measure these outcomes and instruments’ validation processes [16]. In this study, we also assessed patient satisfaction with the multimedia PtDA for the intervention group.

In summary, Patient Knowledge Recall about the PICC Procedure Survey included 19 multiple choice and true or false questions and was developed on the basis of recent PICC clinical practice guidelines [16] and patients’ information needs about the procedure indications, benefits, contraindications, insertion site, complications with their probabilities (less common, common, and rare risk factors), and patient and health care team roles in the care and safety of a PICC [16]. The same survey was used to measure knowledge retention.

Patient Satisfaction with the PICC Informed Consent Process Survey was created on the basis of the AHRQ’s Guide for Informed Consent; it included 10 items with a 5-point Likert-type scale of agreement [16]. The survey also asked patients about their overall satisfaction with the informed consent process using a 5-point Likert-type scale that ranged from 5 (very satisfied) to 1 (very unsatisfied). For the 2 study groups, the survey measured patient satisfaction with the information provided by the vascular access team. For the intervention group, this discussion took place after watching the multimedia PtDA for the PICC.

Patient Satisfaction with the Multimedia PtDA for the PICC Survey was created on the basis of the AHRQ’s Patient Education Materials Assessment Tool Guide for Audio and Video Materials and the Criteria for Effective Patient and Consumer Education Materials [19,21] and included 14 items of a 5-point Likert-type scale of agreement. The survey was followed by a question that measured overall patient satisfaction with the PtDA (a 5-point scale) and 3 other questions that measured the number of times the patient watched the PtDA, whether the patient thinks he or she will watch the program again later, and additional comments. In all surveys, we used the term “video” instead of “multimedia PtDA” to promote patients’ understanding of the items because the term “multimedia” is not frequently used by the public.

### Study Procedure

Four nurse educators from the ACUs and ICUs administered the surveys. During the study period and at the beginning of every working shift, the vascular access team provided the nurse educators with a list of all PICC orders. The list included the patient name, medical record number, room number, and the unit. After obtaining the consent form, the vascular access team called the nurse educators informing them about the time they obtained the consent form. The nurse educators approached the patients on the list who completed signing the consent form, discussed the study purpose, and emphasized voluntarily participation and patients’ rights to withdraw from the study at any point. Owing to a total of 4 surveys in this study to measure the main study outcomes, the nurse educators administered 2 surveys at a time for each patient to improve the response rate and decrease the burden on our patients. For example, Knowledge Recall about the PICC Procedure and Patient Satisfaction with the Multimedia Program surveys were administered 4-8 hours after obtaining the consent form, while Knowledge Retention and Patient Satisfaction with PICC Informed Consent Process surveys were administered 24-36 hours after obtaining the consent form. Overall, 6-7 surveys were collected from each of the 10 inpatient units where a PICC was ordered to reach the desired sample size for each study phase. In addition, the medical record number was used to connect patients’ responses on all surveys. Furthermore, we collected the following patient demographic data from the EMR and patients when the information was not available in the EMR: gender, age, race, education level, preferred language, time of admission, date of admission, and the unit.

The surveys were administered in the English language to all patients, except for 2 patients who indicated Spanish as their preferred language. For these 2 patients, a certified interpreter translated the survey questions.

## Results

### Patient Characteristics

All patients provided complete responses to all questionnaires, except for 2 patients in the postintervention phase who were excluded from the analysis. We observed no differences in patient characteristics between the control and intervention groups based on chi-square statistic (*P*>.99; Table 1). Patient age ranged from 24 to 94 years in the control group and 23 to 77 years in the intervention group, and they had similar mean age (mean 51.7 [SD 17.1] vs mean 51.9 [SD 14.3]; *P*=.90). The majority of patients were white and Hispanic individuals (Table 1). Other races included black (4 in the control group and 4 in the intervention group), American or Alaskan Native (1 in the intervention group), and Asian (1 in the control group). All patients watched the English version of the PICC multimedia PtDA, except for 2 patients who watched the Spanish version (Table 1).

### Knowledge Recall and Knowledge Retention

**Figure 1:**
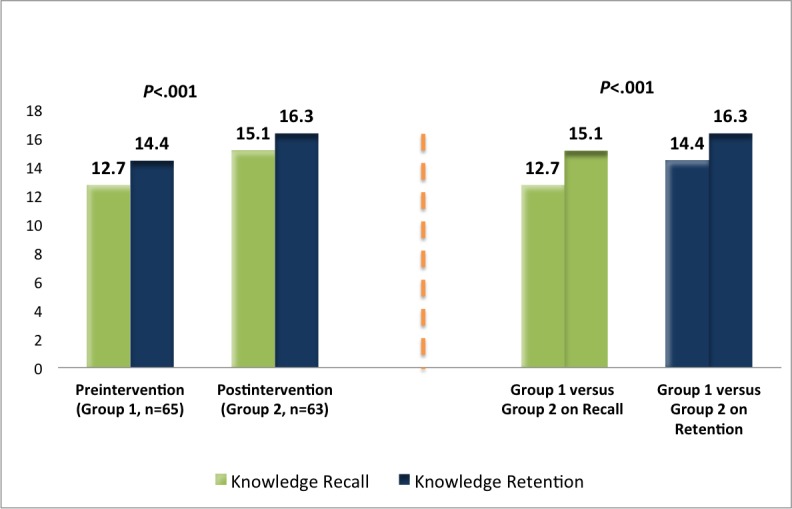
Mean difference of correctly answered questions in knowledge recall and retention surveys (19 questions) for the control (n=65 patients) and intervention (n=63 patients) groups.

Patients’ scores on knowledge recall and knowledge retention ranged from 6 to 18 in the preintervention phase and 9 to 19 in the postintervention phase (out of 19 points). Although knowledge retention was higher than recall at the group level, in general, the 2 study groups scored above average on both surveys (Figure 1). In comparison to the control group, the intervention group scored around 2 points higher on knowledge recall (*t*_*125*_=4.9, *P*<.001) and knowledge retention (*t*_*126*_=4.8, *P*<.001; Figure 1).

**Table 1:** Patient characteristics (N=128).

Characteristic		Control group (n=65)	Intervention group (n=63)	*P* value
**Age in years, n (%)**				**.41**
	<30	11 (17)	5 (8)	
	30-50	22 (34)	22 (35)	
	51-65	18 (28)	23 (37)	
	>65	14 (22)	13 (21)	
Male, n (%)		28 (43)	34 (54)	.20
**Race, n (%)**				**.92**
	Hispanic	30 (46)	25 (40)	
	White non-Hispanic	30 (46)	33 (52)	
	Others	5 (8)	5 (8)	
**Level of education^a^, n (%)**				**.07**
	Illiterate	1 (2)	1 (2)	
	Primary education to less than high school	4 (6)	7 (11)	
	High school	38 (58)	40 (63)	
	College or bachelor	21 (32)	11 (17)	
	Graduate	1 (2)	4 (6)	
**Language of consent^b^, n (%)**				**N/A^c^**
	Spanish	1 (2)	1 (2)	
	English	64 (98)	62 (98)	

^a^The 3 groups used to perform the chi-square test were high school, college or bachelor, and others. The “others” included all other groups under the level of education because of the small sample size under those categories.

^b^The chi-square test was not performed because of the obvious lack of significance between the categories related to similar frequencies under each category and the small cell size under Spanish.

^c^N/A: not applicable.

### Patient Satisfaction With the Multimedia Program

Table 2 presents the mean agreement (patients who reported agree or strongly agree) of patient satisfaction with the multimedia PtDA for the PICC procedure for patients in the intervention group. All patients were highly satisfied with the multimedia PtDA with a mean score of >4.5 out of 5 on all items. Items with the highest mean scores were related to the effect of the multimedia PtDA on knowledge retention, patient readiness to learn, and complete understanding of procedure complications and patient role in maintaining the safety of the PICC. Of all patients, 17% (11/63) watched the PtDA program twice, 83% (52/63) watched it once, and 57% (36/63) mentioned that they would like to watch it again. The overall mean patient satisfaction with the multimedia PtDA was 4.6 (SD 0.6).

### Patient Satisfaction With the Informed Consent Process

Table 3 presents the mean agreement (patients who reported agree or strongly agree) of patient satisfaction with the informed consent process for the control and intervention groups at the item level. Patients in the control group rated their satisfaction with the discussion between them and the nurse from the vascular access team. Patients in the intervention group rated their satisfaction with the discussion between them and the nurse from the vascular access team after they watched the PtDA program. Patients in the 2 groups were highly satisfied with the process with a reported mean of 4.5-5 out of 5 points on each item. No significant differences at the item level were found between the 2 groups (*P*>.99).

In this study, 15% (10/65) patients in the control group reported that the following were omitted from the discussion: patient role in the safety of the PICC (5 patients), provider role in the safety of the PICC (4 patients), other treatment options (4 patients), and common side effects (6 patients). Furthermore, 2 patients commented that they were not ready to engage in the discussion.

Overall, patient satisfaction with the process was high for both the groups (mean 4.8 [SD 0.6] versus mean 4.8 [SD 0.5] for the control and intervention groups, respectively, *P*>.99).

**Table 2:** Satisfaction with the multimedia decision aid program for the peripherally inserted central venous catheter (PICC) for the intervention group (n=63 patients).

Item	Mean (SD)
1. The video better helped me remember the information about this procedure	4.9 (0.2)
2. The video allows me to listen to information when I am ready to do so	4.8 (0.5)
3. After watching the video, I completely understand the common complications of this procedure and know when to report them	4.8 (0.4)
4. After watching the video, I understand my role as a patient in maintaining the safety of the PICC line	4.8 (0.5)
5. The information in the video was comprehensive to include the following: Reasons for PICC Steps of the procedure Common side effects Other treatment options Definition of PICC Patient role in care and safety of PICC Provider role in care and safety of PICC	4.7 (0.6)
6. There was almost no disruption during watching the video	4.7 (0.8)
7. Visual aids (eg, showing the PICC line) in the video were helpful	4.7 (0.6)
8. The video was very beneficial to learn about the procedure	4.7 (0.6)
9. The video allows me to listen to information as many times as I need	4.7 (0.7)
10. The information in the video was clear	4.6 (0.7)
11. The information in the video was easy to understand	4.6 (0.8)
12. Speed of presenting the information in the video was reasonable	4.6 (0.8)
13. I highly recommend this video to supplement the consent process for PICC	4.6 (0.8)
14. I feel the video decreased my level of anxiety	4.6 (1.0)

**Table 3:** Patient satisfaction with the informed consent process (N=128).

Item	Mean (SD)	
	Control group (n=65)	Intervention group (n=63)
1. Provider attitude during the discussion was positive	5 (0)	4.9 (0.1)
2. Speed of information provided was reasonable	4.9 (0.4)	4.9 (0.1)
3. Disruption during the discussion was minimal	4.8 (0.5)	4.8 (0.6)
4. I completely understand the common complications of this procedure and know when to report them	4.8 (0.5)	4.9 (0.4)
5. The information provided by the nurse was clear	4.7 (0.3)	4.9 (0.3)
6. The information provided was easy to understand	4.7 (0.9)	4.8 (0.4)
7. Timing of the discussion was convenient	4.7 (0.6)	4.9 (0.4)
8. I understand my role as a patient in maintaining the safety of the peripherally inserted central catheter (PICC)	4.6 (1)	4.9 (0.4)
9. The nurse answered all of my questions sufficiently	4.6 (0.7)	4.9 (0.3)
10. The information provided was comprehensive to include the following: Definition of the PICC Reasons for the PICC Steps of the procedure Common side effects Other treatment options Patient role in care and safety of the PICC Provider role in care and safety of the PICC	4.5 (0.8)	N/A^a^

^a^N/A: not applicable.

## Discussion

### Principal Findings

This study examined the effectiveness of a multimedia PtDA to supplement the consent process of a PICC for patients in the ACUs and ICUs on knowledge recall, knowledge retention, and patient satisfaction with the informed consent process and satisfaction with the PtDA. Patient-centered care and shared decision-making models are based on empowering patients with sufficient, clear, and easy-to-understand information about treatment procedures. Complex care environments are one of the barriers to effective informed consent process even for nonurgent procedures such as a PICC due to time pressure, workload, complexity of medical conditions, special patients’ circumstances, and diverse patients’ needs. Research studies support the limitations of the conventional informed consent process where providers do not allocate sufficient time to discuss procedures’ details or provide a meaningful dialogue [1,2]. Failure to apply these elements in a consent process would result in signing a piece of paper for record keeping rather than informing and engaging patients in care processes. Furthermore, well-designed multimedia PtDAs are effective tools to supplement the consent process of medical procedures in complex care environments.

In this study, patients in the control and intervention groups had a higher level of knowledge retention about the procedure in comparison with knowledge recall; this can be related to the fact that when nurse educators collected the data from patients, although they recorded patients’ answers (whether right or wrong), they corrected the patients’ misunderstanding about the procedure indications, complications, side effects, and patient and provider roles in safety of the procedure when patients provided wrong answers. Nevertheless, patients in the intervention group significantly achieved a higher level of knowledge recall and knowledge retention, supporting the effectiveness of this tool in supplementing the consent process. In addition, patients in both the groups reported high levels of satisfaction with the informed consent process. Patient satisfaction with the multimedia PtDA for patients in the intervention group was overwhelmingly positive.

Some patients in the control group indicated that the consent process missed discussing different aspects of the procedure such as patient and provider roles in the safety of the procedure, common side effects and complications, and other treatment options. In a previous study, patients reported missing information in the consent process when, in fact, this information was discussed by the health care team [16]. This supports the need for a self-paced resource, such as a multimedia PtDA, accessible to patients when needed to review the information discussed with them.

Our multimedia PtDA for the PICC provided complete information about the procedure. In addition, the PtDA focused on the patient role in the safety of the procedure, an area that is often ignored in consent processes. All surveys also included items that reflect patient role in the safety of the procedure. Although our patients in the 2 groups scored high on these items, one of our patients in the intervention group stopped a nurse from touching his PICC line and asked her to wash her hands based on what he saw in the video.

Hand hygiene is the number one strategy to prevent CLABSIs. CLABSIs are ranked the most common and the most costly hospital-acquired infections (account for US $46,000 per event [22]), and they result in thousands of deaths each year [23]. CLABSIs are “never event” that should never happen if appropriate preventable measures are in place such as hand hygiene and empowering patients to understand and immediately report side effects and complications. In the future, it would be interesting to examine the effect of the multimedia PtDA on CLABSI incidence rate.

The multimedia program was meant to be a self-paced learning tool that takes into consideration patient readiness to engage in care processes. Some of our patients in the control group mentioned that they were not ready for the discussion. Readiness to learn was ranked the second highest item by the intervention group in the patient satisfaction with the multimedia program survey. Many patients in the intervention group watched the video for more than once and indicated their interest to watch it again. The availability of such resource to the patients is necessary to reinforce knowledge and engagement in care processes.

### Limitations

This study has the following 2 limitations. First, although the ratios of our Hispanic and white patients were similar to the patient populations we see at the hospital level, almost all of our Hispanic patients who participated in this study indicated English as their preferred language for the discussion and the PtDA. In future research, we need to focus on engaging a larger sample of Hispanic population who would use the Spanish version of the program to test whether the Spanish version has the same positive effect as the English version. Second, the nurse educators who served as data collectors were instructed to correct the wrong responses provided by patients in the knowledge recall and retention questionnaires after recording the original patient responses. Although this reflects ethical and professional practice principles to maintain safety, it could have biased our knowledge retention scores. Nevertheless, the effectiveness of the PtDA was supported by other results such as (1) the significantly higher knowledge recall scores for the intervention group than for the control group; (2) the high agreement scores assigned to the “readiness to learn,” “complete understanding of procedure complications,” and “complete understanding of the patient role in maintaining the safety of the PICC” items by the intervention group in the patient satisfaction with the multimedia program survey; and (3) the availability of the PtDA as a self-paced learning tool supported by watching the video for more than two times by some patients.

### Conclusions

This study reveals that the multimedia PtDA is an effective standardized, structured, and self-paced learning tool to supplement the consent process of the PICC and improve patient satisfaction with the process, knowledge recall, and knowledge retention.

## Abbreviations

ACU: acute care unit
AHRQ: Agency for Healthcare Research and Quality
CLABSI: central line-associated bloodstream infection
EMR: electronic medical record
ICU: intensive care unit
PICC: peripherally inserted central venous catheter
PtDA: patient decision aid
